# How Much Progress Have We Made? Trends in Disparities in Tobacco Use

**DOI:** 10.5888/pcd17.200090

**Published:** 2020-09-17

**Authors:** J.H. Kingsbury, J. D’Silva, E. O’Gara, M.J. Parks, R.G. Boyle

**Affiliations:** 1 Minnesota Department of Health, St. Paul, Minnesota; 2 ClearWay Minnesota, Bloomington, Minnesota; 3 University of California, Office of the President, Oakland, California

## Abstract

**Introduction:**

Reducing tobacco-related health disparities has been a public health priority for more than 2 decades, yet disparities in cigarette use have remained steady or worsened. Less is known about how disparities in other tobacco products have changed over time. Our study examined trends in cigarette and other tobacco product use in Minnesota with the goal of informing efforts aimed at reducing disparities.

**Methods:**

We examined tobacco use disparities as a function of education, income, and race across the Minnesota Adult Tobacco Survey results in 2010 (N = 7,057), 2014 (N = 9,304), and 2018 (N = 6,055). Tobacco use was captured by assessing past 30-day use of 4 tobacco products: cigarettes, cigars, e-cigarettes, and smokeless tobacco, plus combustibles (ie, cigarettes and/or cigars) and any tobacco (ie, use of any of the 4 products).

**Results:**

At each wave, those with lower income and education reported greater use of cigarettes, combustibles, and any tobacco than those with higher income and education. Black respondents were more likely to report cigar and combustibles use than White respondents in 2018, whereas White respondents were more likely to report smokeless tobacco use in 2014. We saw no significant wave-by-demographic interactions, suggesting that the magnitude of the disparity remained unchanged over time for any tobacco product.

**Conclusion:**

Substantial disparities in tobacco use remain across education, income, and race, even in a state such as Minnesota with a strong tobacco control program. Additional efforts are needed to close disparity gaps and reach endgame tobacco use targets for all subpopulations.

**Summary**

**What is already known on this topic?**

Recent evidence has shown that disparities in cigarette use across demographic groups remain unchanged or have worsened.

**What is added by this report?**

Our study replicates these findings for cigarettes and reveals a similar pattern for other tobacco products whereby disparities in use of e-cigarettes, cigars, and smokeless tobacco have not changed in the past 8 years, even in a state with comprehensive smoke-free laws and high tobacco taxes.

**What are the implications for public health practice?**

Tobacco control professionals and policy makers must prioritize equity with any proposed strategies or policies to reduce persistent disparities and achieve tobacco-free societies.

## Introduction

Although cigarette smoking has decreased substantially in the United States over the past several decades (1), declines in prevalence have been slower for some communities (2). Smoking disparities remain unchanged (3,4) or have increased (5,6) for several groups who are targeted by the tobacco industry. Many racial and ethnic minority groups smoke at higher rates than the national average (4,7), as do people who identify as belonging to sexual and gender minority groups (7) and those with lower socioeconomic status (8,9). At the same time, some subpopulations are at or approaching endgame levels of cigarette use (<5%). For example, only 4.1% of those with a graduate degree report current cigarette use compared with 23.1% of those with less than a high school diploma (1). Further efforts are needed to achieve low smoking prevalence across all subpopulations.

Cigarettes remain both the most deadly and commonly used commercial tobacco product (1); however, many other products (eg, e-cigarettes, cigars) have gained in popularity (10,11), and less is known about disparities in use of these products. Promotion by the tobacco industry (10) has successfully led to increases in the use of some tobacco products. For instance, e-cigarette use has increased dramatically among youth and young adults in the past 5 to 8 years (1,12), and the use of cigars has grown in the past 2 decades among non-Hispanic Black adults (13). Understanding whether trends are changing across all tobacco products is an increasingly important issue given that current tobacco control efforts have reported little success in reducing tobacco use disparities overall (3–6). Furthermore, if groups that experience tobacco-related disparities (eg, low income/education, racial/ethnic minorities) continue to use combustible products while those with high incomes and education and White smokers quit or switch to less harmful products, disparities in tobacco-related health outcomes could be exacerbated.

Although reducing disparities has been a public health priority for more than 2 decades (14) and some promising frameworks have been identified (eg, the social determinants of health approach) (8), more work is needed to elucidate effective approaches for reducing tobacco-related disparities at the state and national level. Examining tobacco use data from states with strong tobacco control programs may help inform efforts to reduce tobacco-related disparities, because these locations may offer an environment that is more conducive to equitable progress in tobacco control.

Our study examines trends in cigarette and other tobacco product use to measure progress in reducing tobacco-related disparities. This is especially relevant in Minnesota, a US state with comprehensive and long-term tobacco control programs. For more than 2 decades, Minnesota has taken actions to address tobacco use through banning indoor smoking, increasing tobacco taxes, providing free cessation services, and implementing other norm-change initiatives — efforts that have helped decrease adult smoking prevalence by 35% since 1999 and youth smoking prevalence by 70% since 2000 (15). Despite these efforts, previous research has identified gaps in the effectiveness of changing statewide cigarette smoking rates across all communities (6). We sought to provide estimates of other tobacco product use and examine changes in use over time by using data from 3 rounds of the Minnesota Adult Tobacco Survey (MATS) (16).

## Methods

**Data**. We used data from the 2010, 2014, and 2018 MATS, a statewide, cross-sectional telephone survey that assessed tobacco use among Minnesotans aged 18 or older (2010, N = 7,057; 2014, 9,304; and 2018, 6,055). The survey used a landline and cellular telephone random-digit-dial sampling method. Sampling consisted of a 2-step process: a household screening questionnaire to identify households, followed by sampling within the household. Combined response rates ranged from 71% to 73% across the 3 waves. Data were weighted to account for sampling and geographic stratification, ensuring statewide representativeness. Additional information on MATS is available elsewhere (16). This study was approved by the Minnesota Department of Health Institutional Review Board.

**Sample**. We examined overall prevalence and differences by education and income for the entire study sample at each wave. Analyses examining race were limited to respondents who self-reported race as Black/African American or White.

**Measures**. Tobacco use was measured as use in the past 30 days of 4 tobacco products: cigarettes, cigars, e-cigarettes, or smokeless tobacco. Respondents reporting that they had used a particular product at least once in the past 30 days were categorized as users of that product. Combustible tobacco users were those reporting any past use in the past 30 days of cigarettes, cigars, or both; any tobacco users were those reporting use of any of the 4 tobacco products in the past 30 days.

Tobacco use disparities were examined as a function of education, income, and race. Education and household income were transformed into categorical variables (0 = high school diploma, general equivalency diploma, or less [low education]; 1 = some college or more [medium/high education]) and low income (0 = ≤$25,000/y) versus medium/high income (1 = >$25,000/y). Those with low income and/or education historically reported higher tobacco use than those with medium/high income and education, so we were particularly interested in examining use in these groups. Race was assessed via a single question with 6 response options (White, Black, Asian, Native Hawaiian/Pacific Islander, American Indian/Alaskan Native, other). Analyses examining racial disparities focused on White and Black respondents because of sample size.

**Analysis**. Weighted prevalence for each tobacco product and category of use (combustible, any use) was calculated at each wave for the entire sample, low versus medium/high education, low versus medium/high income, and Black versus White race. We used weighted logistic regression to test for demographic differences at each wave. These analyses used dummy variables for education, income, Black or White race, and survey wave. The reference group for the survey wave variable was alternated to test for differences over time (ie, 2010–2014, 2014–2018, 2010–2018). An interaction term (ie, demographic-by-survey wave) was used to determine whether differences in each demographic variable changed over the 3 waves. All analyses were conducted in SPSS version 24 (IBM Corp).

## Results

Cigarettes were the most commonly used tobacco product at each wave (17.3% in 2010, 15.5% in 2014, and 15.3% in 2018), followed by smokeless tobacco in 2010 (4.0%) and by e-cigarettes in 2014 (5.9%) and 2018 (6.0%)( Table). 

**Table:** Prevalence of Tobacco Use Over Past 30 Days by Demographic Characteristics Across Survey Wave, Minnesota Adult Tobacco Survey (MATS)^a^

Characteristic	2010 (N = 7,057)	2014 (N = 9,304)	2018 (N = 6,055)
**Cigarettes**			
**Overall**	17.3 (16.1–18.5)	15.5 (14.5–16.5)	15.3 (14.1–16.6)
**Race**			
Black	26.7 (18.3–37.1)	23.3 (17.2–30.7)	23.2 (16.9–31.1)
White	16.5 (15.3–17.8)	14.9 (13.8–16.0)	14.7 (13.4–16.1)
**Annual income, $^b^**			
≤25,000	30.0 (26.4–33.8)	26.6 (23.6–29.9)	30.4 (25.9–35.2)
>25,000	14.8 (13.6–16.2)	13.7 (12.5–14.9)	12.7 (11.4–14.1)
**Education^c^**			
≤High school diploma or GED	23.1 (20.7–25.7)	22.4 (20.3–24.6)	23.3 (20.6–26.3)
≥Some college	14.0 (12.8–15.4)	11.9 (10.9–13.1)	11.5 (10.3–12.9)
**Cigars**			
**Overall**	3.2 (2.7–3.9)	2.9 (2.5–3.5)	3.0 (2.4–3.7)
**Race**			
Black	6.9 (3.0–14.8)	5.8 (3.0–10.9)	7.0 (3.7–13.1)
White	3.2 (2.7–3.9)	2.8 (2.3–3.3)	2.8 (2.2–3.5)
**Annual income, $^b^**			
≤25,000	4.0 (2.6–6.1)	3.1 (2.1–4.7)	4.7 (2.9–7.6)
>25,000	3.3 (2.7–4.0)	3.0 (2.5–3.6)	2.7 (2.1–3.4)
**Education^c^**			
≤High school diploma or GED	4.0 (2.9–5.3)	3.0 (2.2–4.0)	4.2 (2.9–5.9)
≥Some college	2.9 (2.3–3.5)	2.9 (2.4–3.6)	2.4 (1.9–3.0)
**E-Cigarettes**			
**Overall**	0.7 (0.4–1.1)	5.9 (5.3–6.7)	6.0 (5.2–6.9)
**Race**			
Black	1.4 (0.2–9.2)	5.3 (2.7–10.3)	5.1 (2.4–10.4)
White	0.7 (0.4–1.0)	5.8 (5.1–6.5)	5.8 (4.9–6.8)
**Annual income, $^b^**			
≤25,000	1.4 (0.7–3.1)	8.5 (6.7–10.8)	8.5 (6.2–11.4)
>25,000	0.5 (0.3–0.9)	5.5 (4.8–6.4)	5.3 (4.4–6.3)
**Education^c^**			
≤High school diploma or GED	1.3 (0.7–2.4)	7.6 (6.3–9.1)	8.2 (6.5–10.4)
≥Some college	0.4 (0.2–0.7)	5.1 (4.4–5.9)	4.8 (4.0–5.8)
**Smokeless Tobacco**			
**Overall**	4.0 (3.4–4.7)	3.6 (3.1–4.1)	3.2 (2.6–3.8)
**Race**			
Black	1.0 (0.2–4.9)	0.4 (0.1–1.5)	0.9 (0.2–3.7)
White	4.2 (3.5–4.9)	3.9 (3.4–4.5)	3.6 (3.0–4.4)
**Annual income, $^b^**			
≤25,000	3.6 (2.3–5.5)	3.5 (2.4–5.1)	1.5 (0.8–3.1)
>25,000	4.3 (3.6–5.1)	3.8 (3.3–4.5)	3.7 (3.0–4.6)
**Education^c^**			
≤High school diploma or GED	4.7 (3.6–6.2)	4.5 (3.6–5.7)	3.6 (2.6–5.1)
≥Some college	3.6 (3.0–4.4)	3.1 (2.6–3.7)	3.0 (2.4–3.7)
**Combustible Tobacco (Cigarettes and/or Cigars)**			
**Overall**	18.8 (17.5–20.0)	16.9 (15.9–18.0)	16.9 (15.6–18.3)
**Race**			
Black	28.2 (19.6–38.8)	25.1 (18.8–32.6)	26.7 (20.0–34.7)
White	18.1 (16.9–19.4)	16.4 (15.3–17.5)	16.4 (15.0–17.9)
**Annual income, $^b^**			
≤25,000	30.7 (27.1–34.6)	27.6 (24.5–30.9)	31.3 (26.6–35.9)
>25,000	16.6 (15.3–18.1)	15.4 (14.2–16.6)	14.6 (13.3–16.1)
**Education^c^**			
≤High school diploma or GED	24.2 (21.8–26.8)	23.4 (21.3–25.7)	24.8 (22.0–27.8)
≥Some college	15.7 (14.4–17.1)	13.7 (12.6–14.9)	13.3 (12.0–14.7)
**Any Tobacco**			
**Overall**	21.2 (19.8–22.4)	20.5 (19.4–21.7)	21.4 (20.0–22.9)
**Race**			
Black	28.4 (19.8–39.0)	27.6 (21.1–35.2)	27.4 (20.7–35.4)
White	20.6 (19.3–21.9)	20.1 (18.9–21.3)	20.9 (19.4–22.5)
**Annual income, $^b^**			
≤25,000	31.7 (28.1–35.6)	31.3 (27.9–34.4)	34.4 (29.9–39.3)
>25,000	19.4 (18.0–20.9)	19.2 (17.9–20.5)	19.4 (17.8–21.0)
**Education^c^**			
≤High school diploma or GED	26.7 (24.2–29.4)	27.4 (25.2–29.8)	29.9 (26.9–33.0)
≥Some college	18.0 (16.6–19.4)	17.1 (15.9–18.4)	17.3 (15.8–18.9)

Abbreviation: GED, general equivalency diploma.

a Values are percentage (95% CI). Estimates represent tobacco use over the past 30 days and thus may differ from previously reported current tobacco use estimates from MATS (16).

b Low income (≤$25,000); medium or high income (>$25,000).

c Low education (high school diploma, general equivalency diploma, or less); high education (some college or more).

### Changes in use over time

Logistic regression analyses showed a significant decrease in cigarette use from 2010 through 2014 (odds ratio [OR] = 0.88; 95% CI, 0.78–0.98) and from 2010 through 2018 (OR = 0.87; 95% CI, 0.76–0.99). Combustible product use decreased significantly from 2010 to 2014 (OR = 0.88; 95% CI, 0.79–0.99), and a significant increase occurred in e-cigarette use from 2010 to 2014 (OR = 8.97; 95% CI, 5.59–14.37) and between 2010 and 2018 (OR = 9.06; 95% CI, 5.60–14.68). All other comparisons were nonsignificant.

### Changes in demographic characteristics within wave 

**Income. **Respondents with low income reported higher prevalence at each wave than those with medium/high income (ORs = 2.18 in 2010, 1.90 in 2014, 2.42 in 2018). There were no differences in cigar use by income at any wave. Respondents with low income reported more e-cigarette use in 2014 (OR = 1.43; 95% CI, 1.04–1.97), and those with low income reported less smokeless tobacco use in 2018 (OR = 0.36; 95% CI, 0.16–0.77). Those with low income also reported more use of combustible tobacco (OR = 2.00; 95% CI, 1.78–2.16) and any tobacco (ORs = 1.73 in 2010, 1.62 in 2014, 1.79 in 2018) than did those with medium/high income at all waves.

**Education. **Similar patterns were observed for education. Those with low education reported more cigarette use than those with medium/high education at each wave (ORs = 1.53 in 2010, 1.97 in 2014, 2.11 in 2018). A similar use pattern was observed for cigars in 2010 (OR = 1.51; 95% CI, 1.01–2.25), e-cigarettes at all waves (OR = 2.77 in 2010, 1.42 in 2014, 1.79 in 2018) and for smokeless tobacco in 2014 (OR = 1.66; 95% CI, 1.21–2.29). Those with low education reported more use of combustibles (ORs = 1.47 in 2010; 1.80 in 2014; 1.97 in 2018) and any tobacco use (OR = 1.48 in 2010, 1.74 in 2014, 1.97 in 2018) at all waves.

**Race**. We found no significant differences in the use of cigarettes, e-cigarettes, or any tobacco product between Black and White respondents at any wave. Black respondents were significantly more likely to report cigar use than White respondents in 2018 (OR = 2.42; 95% CI, 1.08–5.39). White respondents were more likely than Black respondents to report smokeless tobacco use in 2014 (OR = 0.09; 95% CI, 0.02–0.39), and Black respondents were more likely than White respondents to report combustible tobacco use in 2018 (OR = 1.62; 95% CI, 1.07–2.47).

### Wave-by-demographic interactions

We used logistic regression to test for changes in demographic differences over time. We found no significant wave-by-demographic interactions (wave-by-income, wave-by-education, wave-by-race) for any tobacco product or product category.

## Discussion

Data from statewide surveys in 2010, 2014, and 2018 showed no change in disparities by race, education, or income for the use of cigarettes or other tobacco products. These findings are consistent with previous research demonstrating that disparities in cigarette use have remained largely unchanged since 2002 (3,4) and extend the literature by demonstrating that this pattern is present for other tobacco products (ie, cigars, smokeless tobacco, e-cigarettes) and product categories (ie, combustibles, any tobacco), particularly in a state with comprehensive and long-term tobacco control programs. Because several counties set targets at very low levels of cigarette smoking, our findings highlight 2 key needs: 1) addressing tobacco use other than cigarettes, and 2) devoting more focus to population subgroups that are disproportionately affected by tobacco-related disparities. Additional efforts should go beyond policies that have been effective at reducing overall smoking, and instead use a tailored approach to target other tobacco products and disparately affected communities.

Cigarettes remain the most commonly used tobacco product among adults (1). Consistent with this finding, many tobacco control initiatives are aimed at reducing cigarette use. Our findings highlight the need for future tobacco control initiatives and media campaigns to target other tobacco products (eg, e-cigarettes) (10,11) as patterns of use shift.

Trend analyses indicated that overall cigarette use in Minnesota decreased from 2010 to 2014 but not from 2014 to 2018. These findings differ from national data, which indicate a significant decline over this time period (17). The stalled smoking rate may be due in part to decreases in quit attempts and quit success among smokers in Minnesota (16) and possibly the fading impact of a tobacco excise tax increase from 2013.

Some researchers have employed modeling to identify promising approaches to reducing tobacco-related disparities. For example, a recent study by Combs and colleagues (18) found that many point-of-sale policies (eg, restricting sales of menthol or all cigarettes to tobacco specialty shops) would have the smallest impact on low income and Black populations because these groups are more likely to live in areas with a high density of tobacco retailers. Combining restrictions on menthol cigarette sales with a 2,000-foot retailer-to-retailer buffer would produce a more equitable effect. Golden and colleagues (19) tested the effect of minimum price laws and excise taxes on socioeconomic disparities in smoking. Their models projected that minimum price laws would reduce disparities more than comparable excise taxes (eg, a $2.00 increase in minimum price vs a $2.00 increase in excise tax). These models offer important insights but are most valuable when considered in conjunction with real-world policy evaluations.

Literature reviews may also help identify promising policy approaches for reducing tobacco-related disparities. For example, Brown and colleagues found that price and tax increases had a consistent, positive impact on socioeconomic inequities for adults (20) and youth (21); other policies and interventions had a neutral or negative equity impact. Additional reviews examining equity impact by race, education, geography, sexual orientation, and gender identity are needed to inform the development of pro-equity tobacco control policies.

As many populations move toward very low levels of cigarette smoking, some have advocated for advancing from conventional tobacco control efforts to more aggressive endgame strategies. These hypothetical endgame approaches, such as ending the sale of commercial tobacco (22), have the potential to improve public health broadly and increase equity in communities that are disproportionately affected by tobacco. However, some approaches, while good-intentioned, may have unintended consequences. For example, if the United States moved to reduce the nicotine content in cigarettes to minimally addictive levels but cigars were excluded from nicotine reduction, this may have the unintended effect of exacerbating disparities for Black versus White tobacco users. Moreover, a cigarette-only policy would not address disparities in the use of other tobacco products. To achieve societies that are completely free of commercial tobacco, tobacco control professionals and policy makers must prioritize equity with any proposed strategies and policy initiatives (8).

Ten years after passage of the Family Smoking Prevention and Tobacco Control Act (23), the Food and Drug Administration has yet to exercise its full authority to regulate tobacco products. State- and local-level regulatory efforts will likely be needed for more advanced tobacco policies, such as density policies and those that restrict the sale of flavored (eg, menthol, fruit, candy) tobacco products (24). The best opportunity for advancing equity may be a combination of local, state, and federal actions.

Our study had limitations. The study employed a past-30-day use measure to ensure uniform measurement across tobacco products over time. Use of additional measures that assess current use frequency (eg, every day, some days) would facilitate comparisons with national data (2) and should be employed in future studies. The current study focused on a single US state and may have limited generalizability to other states or countries. The design of MATS limited our examination of racial disparities to 2 racial/ethnic groups. Robust studies examining how tobacco use disparities are changing over time for other racial/ethnic groups would strengthen the literature. The current study did not examine changes in hookah use; future research should assess this emerging product. Research should also consider heterogeneity within racial/ethnic subgroups (eg, African American race, Somali or Oromo ethnicity). In addition, MATS is conducted every 4 years, so is less able to document rapid changes in use patterns. More frequent assessments are recommended for future research to evaluate progress in reducing tobacco-related disparities.

Substantial disparities in cigarette and other tobacco product use remain across education, income, and race, even in a state with a strong tobacco control program. Novel approaches are needed at the state and local levels to address use of multiple forms of tobacco and to close disparity gaps and reach endgame tobacco use targets for all subpopulations.
